# Human animal relationships in *Bos indicus* cattle breeds addressed from a Five Domains welfare framework

**DOI:** 10.3389/fvets.2024.1456120

**Published:** 2024-09-03

**Authors:** Daniel Mota-Rojas, Alexandra L. Whittaker, Ana C. Strappini, Agustín Orihuela, Adriana Domínguez-Oliva, Patricia Mora-Medina, Adolfo Álvarez-Macías, Ismael Hernández-Avalos, Adriana Olmos-Hernández, Brenda Reyes-Sotelo, Temple Grandin

**Affiliations:** ^1^Neurophysiology, Behavior and Animal Welfare Assessment, DPAA, Universidad Autónoma Metropolitana (UAM), Mexico City, Mexico; ^2^School of Animal and Veterinary Sciences, Roseworthy Campus, University of Adelaide, Roseworthy, SA, Australia; ^3^Animal Health & Welfare, Wageningen Livestock Research, Wageningen University & Research, Wageningen, Netherlands; ^4^Facultad de Ciencias Agropecuarias, Universidad Autónoma del Estado de Morelos, Cuernavaca, Mexico; ^5^Facultad de Estudios Superiores Cuautitlán, Universidad Nacional Autónoma de México (UNAM), Cuautitlán, Mexico; ^6^Division of Biotechnology-Bioterio and Experimental Surgery, Instituto Nacional de Rehabilitación Luis Guillermo Ibarra Ibarra (INR-LGII), Mexico City, Mexico; ^7^Department of Animal Science, Colorado State University, Fort Collins, CO, United States

**Keywords:** zebu cattle, human-animal interaction, beef cattle, dairy cattle, animal welfare

## Abstract

The present review has two objectives, the first is to investigate the differences in temperament between *Bos indicus* and *Bos taurus* breeds and determining the effects on production due to positive treatment and to compare this with negative HAR, by using the Five Domain Model as framework. The second objective is to discuss potential strategies to achieve better HAR when working with *Bos indicus* cattle. *Bos indicus* are more reactive and temperamental than *Bos taurus* cattle. When human animal relationships (HAR) are evaluated, *Bos indicus* cattle may react with greater intensity. They may be more likely to develop a negative emotional state, especially in extensively raised *Bos indicus* cattle that are handled only a few times each year. *Bos indicus* cattle can have positive emotional states when they have frequent positive interactions with people. Interactions with people, both positive and negative, would be in the fourth Domain of the Five Domains model. Cattle that are more reactive during handling may also have lower weight gain, even when they have abundant feed. This would be in the first Domain of Nutrition. When cattle are handled in races and corrals, injuries may be more likely to occur. Injuries and bruises would be in the third Domain of Health. Injuries could be caused by either poor handling practices by people or poor handling facilities. Yelling or electric prod use would be examples of poor HAR. Second Environmental Domain issues may be broken facilities or slick, slippery floors that are associated with falls.

## 1 Introduction

Human-animal relationships (HAR) are implicit in daily routine practices on beef and dairy cattle production systems. HAR is known as this dynamic interaction that can elicit both positive or negative outcomes for animals (1, 2). Depending on the type or HAR, positive (e.g., relaxation/attraction/trust) and negative (e.g., fear/distress/aversion) emotional response or valence can be observed (3, 4). However, regardless of its emotional valence, HAR has a substantial effect on the behavior, welfare, health, and animals’ productivity (5). The Five Domains Model (FDM) proposed by Mellor et al. (6) emphasizes the HAR as an element that highly influences the mental state of animals and is associated with the four physical domains (nutrition, physical environment, health, and behavioral interactions) (7).

Most of the research regarding HAR in dairy and beef farms has been performed in *Bos taurus cattle*, describing several benefits due to a positive interaction (i.e., weight gain, and reduction in carcass bruises). Similarly, positive verbal and physical interactions between cattle with stockpersons resulted in higher milk yields and reduced number of steps and kicks during milking (8). Likewise, training in low-stress handling techniques has been shown to reduce significantly the incidence of bruises on cattle from 20 to 1.3% (9).

In contrast to *Bos taurus*, information on *Bos indicus* breeds is limited, particularly when referring to HAR. This is relevant because it is known that *Bos indicus* are more reactive than *Bos taurus* raised in similar conditions, predisposing them to behavioral responses such as escaping attempts, fearfulness, or aggression (10–12). Other authors have established that the reactivity and more excitable behavior of *Bos indicus* cattle make handling more difficult than *Bos taurus* animals (13, 14). The gregarious nature of *Bos indicus* breeds makes them susceptible to social stress when they are exposed to new environments (e.g., the presence of a new handler inside the pens), showing more intense antipredator responses than *Bos taurus* breeds (15, 16). Moreover, the carcass quality, average daily weight gain (ADG), body condition scores, and endocrine parameters of *Bos indicus* cattle are also affected by animals’ responses to social interaction (e.g., calm, restless, or nervous) (14, 16).

This suggests that routine handling techniques performed in *Bos taurus* might not be suited for *Bos indicus* cattle (17–19). This makes a positive HAR relevant for these breeds to improve the welfare of cattle and prevent risks to both handlers and animals. Due to the reactivity attributed to these breeds, handling of *Bos indicus* animals and exposure to stressors (e.g., novel environments, the presence of unfamiliar people, and changes to their social structure) can be challenging and make them susceptible to negative HAR when stockpeople shout, push, or hit to force them to enter or move through the facility.

The present review has two objectives, the first is to investigate the differences in temperament between *Bos indicus* and *Bos taurus* breeds and determining the effects on production due to positive treatment. It will also address the importance of a positive HAR in *Bos indicus*, considering the temperament differences and by using the Five Domain Model as framework. The second objective is to discuss potential strategies to achieve better HAR when working with *Bos indicus* cattle.

## 2 Differences in temperament between *Bos indicus* and *Bos taurus*

The temperament of cattle can be defined as the behavioral response to human interactions (20). Cattle can be described as either having an excitable temperament or having a calm temperament. An animal with an excitable temperament may have a greater fear response during handling compared to a calmer animal (16). A fearful animal is more likely to react aggressively or unpredictably toward stock people (21). This is one cause of a poor HAR with stock people. Some cattle with a low fear calm temperament may have a better HAR with stock people.

Cooke (16) evaluated and compared *Bos indicus*, *Bos taurus*, and their crosses to find differences between calm and excitable animals. Regardless of the breed, excitable animals had higher cortisol concentrations, particularly in crossbred heifers (57.9 ng/mL) when compared to *Bos taurus* heifers (41.8 ng/mL). However, cortisol levels in calm (16.7 ng/mL) and excitable *Bos indicus* steers (19.6 ng/mL) were significantly lower than those reported in calm and excitable *Bos taurus* heifers and cows.

Another aspect to consider is whether the stress response depends only on temperament or is also influenced by the breed of the animal. Fordyce et al. (22) rated the behavior (movement response when handled in a crush and pound) of Brahman cross bullocks and cows and Shorthorn cattle to determine the correlation coefficients between temperament scores of the animals reared in the same extensive conditions. Using a 7-point scale where 1 represented no movement and 7 violent struggles with jumping attempts, it was found that Brahman cattle had higher temperament scores in Brahman cross bullocks and cows when comparing them to (5.54 vs. 4.44 points). Similarly, Hearnshaw and Morris (13) compared *Bos taurus* (Hereford, Simmental, and Friesian) with *Bos indicus* calves (Brahman, Braford, and Africander). Using a 0–5 scale (0 = stands very quietly, offers no resistance and 5 = unmanageable and dangerous), the authors evaluated seven behavioral responses (tail swishing, straining back, backward and forward movements, paddling, escape attempts, kicking, kneeling, and jumping) to obtain an overall temperament score. According to least-square means, *Bos indicus* breeds were more excited, had more abrupt movements (thus, had higher temperament scores: 1.96 vs. 1.05 points) and higher heritability temperament scores (0.46 ± 0.37 vs. 0.03 ± 0.28). Freitas et al. (23) compared the effect of handling in corrals on the stress response of steers and heifers from different *Bos indicus* breeds (Nellore and Guzerá) and *Bos taurus* cattle (Caracu) assessing their entry scores, chute scores, exit score, and FS. The component analysis reported that Caracu animals were less reactive in comparison to Nellore and Guzerá heifers, according to a lower chute score. Nonetheless, the stress response of Caracu heifers was higher than the other breeds when restrained and all animals perceived restrain as a stressor regardless of the breed. In another behavioral study conducted by Piovezan et al. (24), differences between Nellore, Guzerat, Gyr, and Caracu cattle (*Bos taurus*) were reported. The flight time test and behavioral score test was used to measure temperament during weighing (e.g., movement intensity, breathing intensity, vocalization, and kicking). According to the components of variance, it was estimated that Caracu cows had the highest flight time (2.52 ± 1.21 s) while Gyr, Guzerat and Nellore breeds had 1.51 ± 0.97, 1.64 ± 1.18, and 2.14 ± 1.18 s, respectively. Regarding the behavioral score, Caracu cows had the lowest score (1.52 ± 0.83), while *Bos indicus* breeds registered score between 2.48–2.59. This suggest that *Bos taurus* cattle might be easier to handle than *Bos indicus* under the same conditions. Moreover, Fordyce et al. (25) found that *Bos indicus* cross steers that had previous negative experiences with handlers obtain higher bruise scores (3.53) than docile steers (3.03) due to their nervousness. This not only can affect the quality of the carcass but also significantly impairs the welfare of animals. Therefore, using management practices developed for *Bos indicus* is essential to avoid negative effects on performance and welfare (15).

### 2.1 Comparison of temperament between *Bos indicus* breeds

Differences between breeds of *Bos indicus* cattle have also been reported, showing the importance of considering the species and the breed to estimate the social reaction that animals might have towards humans. In particular, behavioral responses such as entry and exit time in the chute (known as the velocity with which the animals enter and exit the chute with pace, trot, or at gallop) as well as chute score and kicking score (frequency of kicking) are used to assess the response of cattle to handling (26). The assessment of the entry time, chute score, kicking score, exit time, and cortisol and glucose parameters at a holding crush, showed differences between Nguni and Boran cattle (*Bos indicus*) (27). Overall, Nguni animals had higher scores (2.61 min, 3.71, 1.90, and 0.35 min, respectively) and cortisol levels in comparison to Boran cattle (1.97 min, 3.24, 1.30, and 0.49 min, respectively) (27), associating these differences to an increased stress response.

When evaluating the exit velocity of *Bos indicus* breeds such as Angus, Braford, Brangus, and Simbrah heifers, no significant differences were observed among breeds. Nonetheless, excitable Braford heifers had less dry matter intake (DMI) per body weight in comparison with the other three breeds (28). Similarly, no differences were found between Braford, Red Brangus, Simbrah, and *Bos indicus* crosses when using chute score. In these animals, the highest temperament score was recorded in Red Brangus cattle (3.78 ± 0.22), while the lowest score was found in *Bos indicus* crosses with 3.46 ± 0.09 (29), showing that *Bos taurus* genetic influence might be playing an important role in crossbreds.

In contrast, Voisinet et al. (30) found non-significant differences in chute score among *Bos indicus* breeds (Braford, Red Brangus, and Simbrah steers and heifers) during weighing and handling. However, gender influenced the behavioral response, finding that heifers (2.23 ± 0.10) were more excitable than steers (1.98 ± 0.10). Therefore, both the breed and the gender could be considered to improve animal handling in *Bos indicus* breeds.

### 2.2 Comparison of variation in *Bos indicus* temperament within the same breed

**Figure 1:**
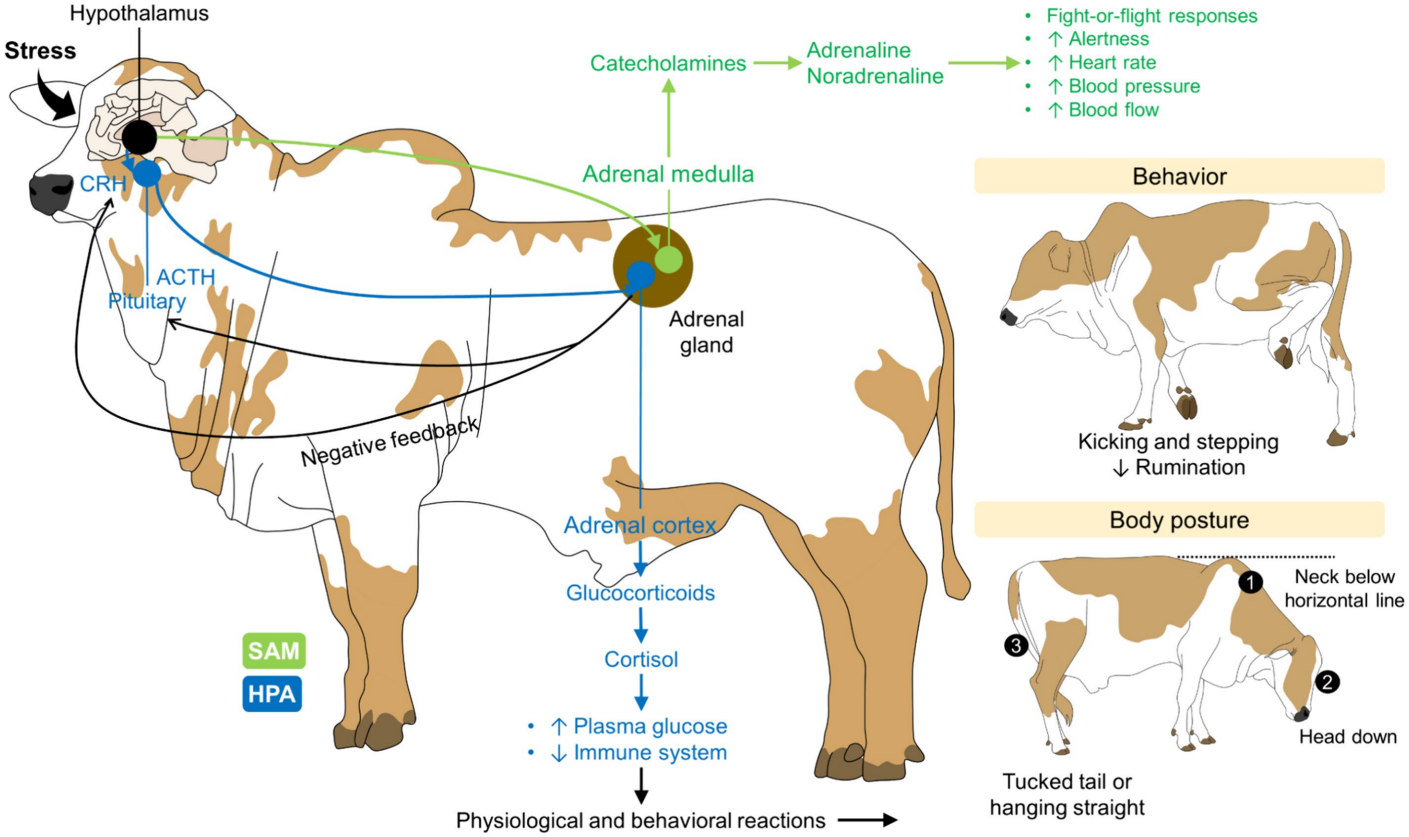
Stress-related axes and their influence on the physiology and behavior of cattle. The perception of a stressor by the nervous system activates the HPA and SAM axes. Both result in the release of glucocorticoids and catecholamines, respectively, to face the challenge and return to homeostasis. Together with the physiological changes such as tachycardia, tachypnea, and increased energy use, behavioral and postural changes also help to identify when dairy cattle perceive an interaction with humans or an event as negative ACTH, adrenocorticotropic hormone; CRH, corticotropin releasing hormone; HPA, hypothalamic–pituitary–adrenal axis; SAM, sympathoadrenal-medullary axis.

Individual differences within *Bos indicus* cattle have also been reported. For example, Cooke et al. (31) assessed the effect that exit velocity has on plasma cortisol concentrations in 170 Nellore heifers. The exit velocity score classified animals as adequate (<3) and excitable (>3). Heifers classified with an adequate response had lower cortisol concentrations (35.8 ng/mL), a greater body condition score (BCS) (6.05), and ADG (0.86 kg/day) than excitable animals (50.8 ng/mL, 5.73, and 0.78 kg/day, respectively). This study suggests that temperament can also affect the health status of cattle, making them more reactive to social stressors and the consequent sympathetic activation and sustained cortisol release—which is an immunosuppressor when chronically released (21, 32, 33). Figure 1 schematizes the stress response and the consequent physiological and behavioral alterations that have been observed in dairy cattle (34–36).

Similarly, Mello et al. (37) determined the reactivity score in Nellore cattle, evaluating the movement, breathing, and exit velocity. On an scale of 3 to 12, <4 = calm and > 9 = very reactive. Highly reactive animals had higher cortisol concentrations than those regarded as calm (54.45 ± 3.39 nmoL/L and 35.04 ± 1.78 nmoL/L, respectively), which can be related to a sympathetic-mediates stress response that might cause physiological consequences. In the same breed, it was found that animals with previous experiences with overly aggressive handling had a greater flight speed (FS) (2.07 ± 1.18 m/s) than gently handled cows (1.74 ± 0.75 m/s) (38). Moreover, other authors have mentioned that age influences the temperament of Nellore cattle (24), where young and calmer bulls adapt better to feedlot conditions (39). The fact that *Bos indicus* animals can be more reactive towards stockpeople also increases the likelihood of aggression by the handler during future management to control the herd (38).

Recently, the genetic parameters for behavioral and growth traits have been studied in Nellore cattle (40), reporting that heritability scores for temperament and FS were 0.08 and 0.12, respectively. These findings support the importance of breeding programs that promote less excitable animals. In other reports, a genetic influence on the temperament of Brahman cattle has been found. When considering an exit velocity of 0.16–1.82 m/s as calm and between 3.05–10.83 m/s as temperamental in both females and males, 14 single nucleotide polymorphisms were associated with cattle temperament scores (41). Thus, considering and preventing events that can make already reactive animals more excitable might decrease the incidence of handling accidents.

### 2.3 Effect of temperament on productivity in both *Bos indicus* and *Bos taurus* cattle

The breed and species, as well as their temperament, can affect the performance of animals, finding that highly excitable cattle can reduce their productivity (42). In this sense, calm and excitable Nellore cattle showed differences regarding the final body weight, ADG, feed efficiency, and meal size, where calm animals had the highest values (492 kg, 1.30 kg/d, and 139 g/kg vs. 463.22 kg, 1.06 kg/d, and 119 g/kg in excitable cattle, respectively) (43). The ADG and DMI in calmer Angus, Brangus, and Simbrah heifers were greater than in excitable animals (1.60 vs. 1.43 kg/d, 9.41 vs. 8.72 kg/d, and 1.32 vs. 1.12 kg/event, respectively) (28).

As previously mentioned, *Bos indicus* cattle might react negatively to human interactions, and this can affect nutrition-related parameters such as body condition score (BCS). This was reviewed by Cooke (16) in 170 weaned Nellore heifers. The author evaluated the association between chute score, exit velocity, and exit scores on ADG, BCS, and cortisol concentration. It was found that excitable *Bos indicus* cattle had a lower ADG (9.3%) and BCS (4.8%) together with cortisol increases of up to 41.89%, in contrast to less excitable cows. In another study, Burrow and Dillon (44) evaluated 96 Brahman x Shorthorn heifers to establish the relation between FS after leaving the weighing crush and growth in a feedlot. The authors observed that animals with higher FS (regarded as highly reactive) had an ADG of 0.79 kg/day, in contrast to calm heifers with an ADG of 0.91 kg/day.

Similarly, Fordyce and Goddard (45) reported a correlation between crush and race test and weight gain (*r* = 0.14), observing that animals that were more reactive to handling had significantly poorer body conditions. This is possibly explained by what was observed by Müller and von Keyserlingk (46) who evaluated the relationship between FS and ADG. They observed that FS increased over time, in addition to a correlation between ADG and FS (*r* = 0.50). Reductions in avoidance distance (AD) after frequent human interactions have been related to a higher body condition in Indian crossbred cows (47). The positive and negative effects of human presence during feeding times could be related to the level of fear in the animals that can influence food consumption and, therefore, weight gain. Although these studies did not focus on HAR, the results suggest that naturally temperamental animals can have adverse reactions to daily practices, including those when human interaction is needed and might be regarded as a negative stimulus.

Sant’anna et al. (48) also established the association between the movement score and FS in Nellore bulls and its effect on meat and carcass quality. Through a linear mixed model, these authors found that reactive animals with higher movement scores (3–5) affected the quality of the meat, having lower meat lightness (36.41 ± 0.26) and yellowness (11.58 ± 0.16), as well as lower hot carcass weight (283.09 ± 3.63 kg) when compared to least reactive bulls. Therefore, temperament traits influences the productivity and reproductive performance of *Bos indicus* cattle (37).

When comparing Brahman and Angus steers and heifers, a higher FS (2.1 ± 0.99–1.5 ± 0.74 m/s) and crush score (2.8 ± 0.73–1.5 ± 0.59) was found when comparing it to Angus cows (2.0 ± 0.62–1.0 ± 0.42 m/s and 1.6 ± 0.64–1.1 ± 0.33, respectively). In *Bos indicus*, highly agitated animals had a reduced feedlot growth rate and feed intake (49). Likewise, similar to *Bos taurus* cattle, reactivity is also used as a predictor of feedlot performance in Nellore and Guzerat calves, where animals with a greater FS reduced their ADG by 0.14 kg/calf per day (50).

León-Llanos et al. (14) observed in 190 Brahman steers (*Bos indicus*) that calmer animals—which might promote easier handling– had greater weights at slaughter (+23 kg) in comparison to nervous animals (Calm = 509.6 kg vs. Nervous = 486 kg). Moreover, in Braford, Brangus, and Simbrah cattle it was found that excitable animals had 25% of carcasses with dark cuts. In contrast, only 6.7% of borderline dark cutting was present in calm animals (30). Similarly, in Braford, Red Brangus, and Simbrah cattle was observed that 40% of highly agitated animals that struggled violently during restraint had a Warner-Bratzler shear (WBS) force above 3.9 kg, while cattle that stood still when restrained had a mean WBS force of 2.86 kg (30). Johnson et al. (51) compared Angus and Brahman steers to evaluate meat and muscle characteristics, finding that Brahman’s WBS was higher than Angus cattle (5.2 vs. 4.1 kg), while the tenderness decreased in the same breed (6.1 in Angus and 5.5 in Brahman). This means that the proteolytic process in muscle can differ between breeds, and this might be associated with the reaction of animals to handling or restraint and the susceptibility to social stress in *Bos indicus* cattle.

Some authors do not report behavioral differences between excitable/calm cattle or between *Bos indicus* and *Bos taurus* breeds (52); however, in dairy systems, a study performed in 72 first-calf heifers of the Black-and-White breed under standard milking showed that calm heifers had a production increase of 14.4% and longer milking time (by 23.4%) than excitable cows, who also reduced their milking cycle by 69.6% (53). The reduced milk yield and milking capacity might be associated with a stronger stress response, as reported by Cooke et al. (54), who determined that more excitable Nellore cattle had higher plasma cortisol levels (+10 ng/mL) than calmer cows.

The reproductive performance may also be affected by the animal’s response to social interactions. Another study evaluated Nellore cows undergoing artificial insemination. Calm animals had the largest diameter of follicles (14.4 ± 0.2 mm) and pregnancy rates at days 30 and 60 (56.4 and 50.0%) than very reactive cows (diameter of 13.2 ± 0.2 mm and pregnancy rates of 51.0 and 42.7%, respectively) (37). Likewise, excitable animals reduced their pregnancy and birth rates by 6.3 and 6.5%, respectively. In Nellore cattle, Rueda et al. (38) associated FS and chute score with pregnancy rates during artificial insemination. It was found that the chance of pregnancy in highly excitable cows was 42.62, 10% less than the one reported in calm animals. This might indicate that excitable *Bos indicus* have a higher chance to react negatively to several management practices inside beef and dairy systems, representing a risk for the handlers as well (20, 55). In this sense, Tirloni et al. (56) reported that handling practices are correlated with reactivity scores of Nellore cattle, meaning that animals become more aggressive when handled with loud noises, kicks and the use of electric prods (reactivity score of 2.12 ± 0.07) than those exposed to low-stress handling (reactivity score of 1.62 ± 0.05).

Grandin and Shivley (57) also found animals’ responses not only dependent on genetic predisposition, but on previous experiences. The literature shows some controversy as to whether the duration of the effect of the interaction is short or long term. Some studies reported that the interaction in early stages between the animal and the human have no long-term effects (58, 59); while Silva (60) found that good practices of handling applied during the pre-weaning period have long-term beneficial effects in crossbred *Bos indicus* x *Bos taurus* heifers’ calves. Moreover, when these good practices were combined with brushing it was observed improved HAR and a reduction in heifers’ fear of humans. If the positive effect of brushing on temperament persists until the onset of their reproductive and productive life of heifers is unknown.

Another aspect that needs to be addressed is the association between positive attitudes toward animals and their effect on animal care. A questionnaire by Bertenshaw and Rowlinson (61) showed that farmers acknowledge that cows can be fearful of humans and that milking temperament is closely related to it. For example, it was reported that farms that used to address cows by their name and provided stress-free environments had higher milk yields (by up to 258 liters). Similarly, when evaluating the flinch/step/kick response, it was found that stock people high on agreeableness and that encouraged positive interaction with cows improved milk yield and milking behavior (62). These studies show that even the attitudes of the farmers can positively influence the HAR.

Therefore, the breed and temperament can affect the adaptability of the animals to new environments as studied by Braga et al. (39) in Nellore young bulls in the feedlot. It was observed that calm bulls had a greater ADG and heavier carcasses than excitable young calves, showing the ability of the animals to positively respond to their environment, handling, and HAR.

## 3 Cattle human animal relationship (HAR) in *Bos taurus* breeds

The HAR has been extensively reviewed in *Bos taurus*. For example, Willson et al. (63) has reported that 70% of handling practices in beef animals include the use of aversive tools such as electric prod or tail twist during unloading, lairage, and in the stun box at an abattoir. This is related to the 27% prevalence of bruises in Holstein-Friesan cows carcasses due to the use of blunt objects and electric prods, as mentioned by Strappini et al. (64). In crossbreed Angus calves, a diminished stress-related response has been found when the animals are handled with minimal stress and prodding (chute score of 1.1), in contrast to those calves exposed to audible records of distressed cattle and noxious noises, together with prodding (chute score of 1.4). de Boyer des Roches et al. (65) observed that fear of people in cows is related to the aggressive or negative attitude of the farmer towards the cow, reporting that only 9.8% of dairy cows accepted being touched by farmers who regularly negatively handle cows. In contrast, this percentage increased when cows interacted with experienced farmers (10.8%) or when they considered cows’ health and HAR as important (10.4%).

In the case of dairy cattle, handling is known to be a significant stressor, to such an extent that cows release the same amount of cortisol when exposed to handling sleeves to be vaccinated, or the milking parlor, as they do when they go to slaughter (66). This is likely because they are unable to differentiate these situations when animal handling is aggressive and causes stress responses in the animals. For example, negative interactions in crossbred *Bos taurus* cows during milking such as hitting increased the respiratory rate, surface temperature, activity level, vocalizations, and defecation (67).

The attitude and behavior of the stockperson towards the cows can also influence the animal’s response and milk parameters. A study carried out by Breuer et al. (68) determined that fearful cows that slowly approached the milker during a fear test produced less milk (<5,632 L/cow/year). Reinforcing this, Rushen et al. (69) reported that the presence of an aversive vs. gentle handler in contact with 14 Holstein cows increased the amount of residual milk (70%), in turn decreasing milk yield (0.72 kg) and increasing heart rate change during milking (73%). Hemsworth et al. (70), evaluated the relationship between the attitude and behavior of stockpersons, and the productivity of cows (predominantly Holstein-Friesian) from 66 commercial dairy farms during lactation. These authors found that the number of forceful negative interactions and unexpected tactile interactions applied by caregivers were negatively correlated with the percentage of cows that approach within 1 m of an experimenter, i.e., the cows were more fearful. This negative attitude and behavior also had a negative impact on production values, such as milk production and percentages of protein and fat (r = −0.36, −0.35 and −0.33 respectively).

Promoting positive HAR through tactile and auditory stimulation has been shown to benefit both humans and animals. Stimulation by stroking the neck, dorsal, and ventral parts of the cows’ bodies can represent positive stimuli if performed properly (71, 72). This was observed in 38 Holstein Friesian cows, which received tactile stimulation by stroking the ventral part of the neck at the milking parlor, 5 min/day for 15 days. Stroking reduced fear-related tachycardia (4). Windschnurer et al. (73) also found that stroking Holstein Friesian and German Red Pied cows during milking –in the morning and evening– by an unfamiliar experimenter for 3 minutes reduced AD from 0.20 ± 0.2777 m to 0.07 ± 0.079 m.

Contrarily to the extensive literature regarding HAR and *Bos taurus* breeds, HAR in *Bos indicus* cattle has limited studies although the excitable temperament of *Bos indicus* is well documented. The following sections will review the literature focusing on HAR in indicus cattle using the Five Domain Model as framework.

## 4 Human animal relationship in *Bos indicus* cattle and the Five Domains

The quality of the HAR has an impact on animal welfare and is closely linked to the Five Domains. As stated by Mellor et al. (6), animal interaction with handlers, owners, veterinarians, or the staff working in dairy milk farms can have both a negative and positive impact on the physical and mental state of cattle. For example, when stockpeople provide adequate nutrition to cattle, proper physical environment, and periodic health revisions to ensure a species-specific behavioral repertoire, both the mental and intrinsic state of the animal can improve but also positive HAR is promoted. When there is a disruption in any of these Five Domains, physiological, behavioral, and emotional alterations might arise (7, 74–77), as increases in cortisol, decreases in oxytocin, increased fear-related behaviors such as kicking, stepping, and large AD, and even changes in the posture and facial expression of cows (78–80).

The influence that a positive/negative HAR has on animal welfare has promoted strategies to understand this issue and implement alternatives (68). An example of this are the training programs aiming to train stockpeople in good practices to properly handle cattle, reducing negative mental states (e.g., fear) and the consequent behavioral and physiological responses to it. Additionally, tactile and auditory stimulation have been shown to be beneficial, such as stroking cattle on different parts of the body, a practice that can reduce AD and fear-related behaviors (71, 80). Likewise, gentle talking by staff can reduce startle reactions and facilitates mustering (2, 81, 82), interaction that will be discussed in the following sections by physical and mental domains.

### 4.1 Animal nutrition and its influence on cattle mental domain

This domain considers the animal’s ability to absorb the nutrients from the food offered. Both the characteristics of the feed and the genetics of the animals play an important role in productive performance and survival (83). An animal with good body condition could be considered an animal with good nutrition provided by the stockperson. By acknowledging the importance of adequate nutrition, farmers prioritize productive parameters but also recognize that this has a crucial role in animals’ physical health that can ultimately affect the mental status of cattle (7, 84–86).

According to Mellor (83), the Nutrition Domain has a close relation with other domains such as the environment and health that drive the animal to display specific feeding behaviors. Feed provision can frequently be a factor in favor of the animal associating the stockperson with something positive (7). However, when the farmer does not provide an adequate quantity and quality of food, the nutritional status of the animal will be affected resulting in a low body condition and, consequently, in hunger, which leads to a negative mental state (87).

Regarding the association between HAR, body condition, and quality of meat products, it has been mentioned that exposure to stressful situations triggers the occurrence of dark, firm, and dry meat. Thus, handling before slaughter must be performed adequately to reduce the level of stress. Furthermore, other events such as transportation, herding, and boarding at slaughter can cause acute fear and affect the quality of the carcass (88, 89). This was reported by Carrasco et al. (90) in 448 *Bos indicus* x European steers. They observed that 81% of the carcasses had at least one bruise and 36.6% had two bruises. From these, 69.6% of the bruises were first-degree (only subcutaneous tissue was affected), and in 69.6 and 44.3% of the cases with hyperglycemia and increases in cortisol (74.7 ng/mL), respectively.

According to what was discussed, indirect interactions such as a lack of attention to the animal’s dietary preferences could limit its nutritional status due to a decrease in consumption. This must be considered to raise *Bos indicus* cattle and prevent affectations on their nutritional status.

### 4.2 Physical environment and how it can affect/benefit animal’s mental domain

The physical environment and facility design can elicit in cattle both negative and positive interactions with humans. It has been seen that cattle move more easily through a race or alley if distractions such as shadows or reflections on shiny metals are removed, or if people up ahead are removed from the animal’s sight (9). This is due to the visual particularities of cattle, having a wide-angle vision but lacking depth perception. Their eye anatomy and number of rod photoreceptors make them see better in low-light environments but will prefer going from a dark to a light place (91–93). Thus, the importance of preventing direct sunlight on their face when moving the animals or adding a light to the entrance of certain facilities to facilitate their movement (2, 94). In other instances, remaining in crowded hallways could elicit fear and consequent stress-related responses (10).

Beef *Bos indicus* cattle are commonly considered to have a more difficult and nervous temperament than *Bos taurus* breeds (13, 22), and the problem appears to be exacerbated by the extensive management practices of the regions that commonly use this kind of breeds (45), which is why the management of these animals as well as the environment must be different, since failure to consider these characteristics can put the animal at risk. For example, Brahman-cross cattle had higher cortisol levels when restrained in a squeeze chute than English crosses (95). This would represent a need to improve management practices in *Bos indicus* cattle to avoid negative association with humans.

Simple changes applied to facilities used for handling cattle, such as adding lighting to driveways or dark corridors, as well as reducing loud and intermittent noises, have all been shown to improve livestock mobility and reduce stress (2). Regardless of the milking system, several stimuli could be perceived as stressor by *Bos indicus* dairy cattle. These include entry to an unknown milking parlor, the presence of unfamiliar stockpersons or the interaction with dominant conspecifics (94). For example, it is known that dairy cattle are afraid of sharp shadows and will balk or react negatively when handled in this environment (94). When animals refuse to move, the use of aversive tools or management (electric prod, tail twisting, and hitting) can occurs in almost the 70% of HAR. This is important because it clearly shows the connection between the conditions of the facilities, the natural behavior and sensory sensitivity, and effects on cattle handling, where easy changes such as removing the visual distractors might ease animal mobilization (96).

Moving cattle within the facility, entering the pen or paddocks, the milking process—whether manual or automatic–, weighing, ear tagging, vaccinating, and blood sampling are known events that might be perceived as negative or stressful by cattle (77, 78, 97, 98), but that can be improved by changing the facility design or training people for gently handling or dairy animals (98–101). Always considering that novelty and strange sights or sounds are often a sign of danger, however in some cases the animals are attracted to novelty (102).

When calves were brought to a new place, they had a lower stress response when the new place looked similar to their home pen (103). A new place that looked very different caused greater stress. It is likely that the visual distractions discussed previously will have the greatest detrimental effect the first time an animal is handled in a new novel facility. Experienced dairy cows will be more likely to willingly walk over a shadow they have seen every day in the milking parlor. The first time a dairy heifer enters the parlor, the same shadow may cause her to stop. Handlers need to be patient and allow the heifer time to stop and look at the shadow. If the new heifer is subjected to shouting, tail twisting, or slaps, she may be more reluctant to enter the milking parlor in the future. It is important to make first experiences with new people, places, or equipment positive (10). Research with rats and sheep indicated that if the first experience with a new place is really aversive, they will be more reluctant to enter it in the future (104).

Pereira et al. (67) reported that the milking system influences the physiological response in the animals. For example, manual milking and putting a bucket below cattle increased negative interactions, such as groping and hitting, also increasing defecation. Mattiello et al. (105) mentioned that the level of mechanization and large farms in the milking parlor increases the frequency of more human-related fear behaviors, due to a lower frequency of contact with stockperson. Which would show that the environment influences the level of fear that the animal experiences in the experimental units. It is also feasible that a poor choice of the design of the facilities can contribute to the presence of negative interactions. Environmental enrichment for Gir x Holstein calves through balls, tires, hanging chains, and scratchers (together with brushing provided by female handlers) showed to reduce lactate concentrations after enrichment (15.66 ± 1.90 mg/dL) and increase oxytocin levels (4.60 ± 0.11 uIU/ml), an endocrine reaction that could be regarded as a positive HAR (106). Likewise, minor changes in the corral and handling practices such as the elimination of bright objects, color contrast, shadows, and dark spots, and eliminating dogs, electric prods, and yelling reduced cortisol values (from 60.40 ± 3.8 to 41.03 ± 2.9 ng/mL) (107). The discussed studies suggest the possible interaction between handling and the environment where the HAR is performed due to the sudden introduction of something novel and new is often stressful (10, 103, 108).

A further consideration in relation to noise is that facility design should also consider environmental noise, for example by use of rubber pads on metal gates. Machinery should be designed to minimize noise and all routine procedures should consider the effect of loud sounds on the animals’ welfare, considering that calm animals are easier to handle, and this reduces the risk of a negative HAR (2). Grandin (2) mentions that visual and auditory particularities of cattle must be considered to provide animals an adequate handling facility that can improve both the HAR and the physical and mental welfare of cattle.

Therefore, providing housing and handling facilities where dairy cattle can be easily handled and moved can also prevent negative HAR and the use of aversive methods such as hitting them with sticks, prods, slaps, or shouting to move animals (109). When designing dairy cattle facilities, is essential to study the behavior of animals to provide adequate and safe environments for the animals that will also be reflected as positive HAR (110).

### 4.3 Physical health

The physical health domain is focused on the capacity for preventive and therapeutic veterinary care in the face of the development of acute and chronic injuries and diseases caused by invasive husbandry practices, surgical procedures and acute, chronic or genetic disorders with effects on the recovery process that give rise to a variety of other negative effects (6, 83),.

Effects on the health of the animals, productive performance and a decrease in live weight, as well as an increase in seizures and disposal of high value cuts due to the presence of bruises, injuries and fractures generated by the use of painful tools during transport was reported (111, 112). The prevalence of bruises and injuries in *Bos indicus* cattle is usually higher than in *Bos taurus* cattle, ranging from 37.5% (113) to 84.5% (114). This is due to limited inappropriate handling and a nervous temperament during mobilization prior to death (14). This is similar to what Vaz et al. (115) found when evaluating carcasses from *Bos indicus* cattle crosses. The highest chance of bruising was found in excitable and in female animals. Moreover, Stanger et al. (116) assessed the phytohemagglutinin-stimulated lymphocyte proliferation in *Bos indicus* steers during 72 h of transport, finding that proliferation decreased making the animals susceptible to other diseases. Handler training programs must consider behavioral responses to challenging situations or temperament to human presence or handling in *Bos indicus* animals.

In dairy cattle, fear or stress during the milking process causes an incomplete emptying of the udder and the accumulation of residual milk due to alteration in the oxytocinergic pathway (69, 117). Therefore, this can be reflected as adverse effects on udder health, increasing the somatic cell counts (SCC) and a higher prevalence of both clinical and subclinical mastitis (118). Additionally, stress and fear have been identified as a determining factor for high SCC, reporting a correlation between human-cow closer voluntary approach and lower SCC (119), being less susceptible to the development of udder pathologies, avoiding the development of mastitis, one of the most common health problems and culling reasons in the dairy industry (120, 121).

The stress level experienced by cattle can have an immunosuppressive effect, making them susceptible to mastitis (122, 123). When considering the differences between *Bos indicus* breeds, Alhussien et al. (124) investigated the combined effect of seasons and lactation stages on mammary immunity in indigenous Sahiwal cows. They observed elevated levels of phagocytic activity and plasma interleukin (IL)-2 during winter and mid-lactation. Furthermore, they observed a positive correlation between plasma concentrations of IL-8, the percentage of milk neutrophils, and the expression of chemokine receptors. According to these results, it is possible to suggest that the excitability of *Bos indicus* must be considered when designing management processes.

Overall, *Bos indicus* females have a lower prevalence of mastitis with respect to *Bos taurus* females. This could be due to the level of intensification and productivity to which they are subjected, without leaving aside the environmental conditions and the sanitary and veterinary practices applied, in addition to the agroecology in which each cow develops. For example, Fesseha et al. (125) observed a higher prevalence of mastitis in Jersey females (78.6%) and Holstein Friesian x indigenous *Bos indicus* cows (51.9%) vs. 16.7% in indigenous *Bos indicus* breeds. However, dairy herds of breeds such as Gyr have a considerable prevalence of subclinical mastitis, directly and negatively affecting milk production and composition (126). In this way, the health of the udder can have an impact on the behavior of cows and predispose negative HAR with the caretakers. Likewise, Shem et al. (127) found that crossbred cattle (*Bos taurus* x *Bos indicus*) had higher prevalence of sub-clinical mastitis (38.3%) than Tanzanian shorthorn *Bos indicus* cows (23.3%), as well as a higher SCC. The authors concluded that this high prevalence may be due to the effect of poor management practices in 84% of the evaluated farms. Factors such as change of milking personnel, technology and environmental and nutritional modifications could lead to a greater risk of infection (mainly in high-performance breeds) (128).

Regarding the minimization of udder health problems, the milking process must consider procedures to minimize the prevalence of clinical mastitis where the milker has influence. For example, the use of gloves during milking, apply post milking teat disinfection, as well as logistical considerations to avoid contamination of automatic milking units (e.g., allocate a specific milking unit for cows with a condition in the mammary gland rinsing, cleaning, and disinfecting the unit after milking) (129). Moreover, Sharma and Phillips (47) found that the health status of Indian cows in shelters is related to AD and to the level of fear towards humans. In their study, 51.2% of the animals allowed to be touched, and 46.6% allowed human approach up to 100 cm. However, reductions in AD were also present in animals with traumatic injuries or lameness due to tarsal joint swelling or ulcerations (r_s_ = between 0.119 and 0.232), probably because animals in pain might not be unable to move away from humans. Therefore, further research is needed to evaluate the effect that health issues might have on the human-animal interaction.

### 4.4 Behavioral interactions

Domain 4 focuses on the interaction of animals with their environment, other non-human animals, and humans, which can be negative, positive, or neutral (6). In the case of *Bos indicus* breeds, the irregular interaction with animals might affect animal handling and make animals susceptible to mistreatment, physical abuse from handlers, or aversive methods to mobilize livestock (130), both for beef and dairy systems. In this sense, human-animal interaction has a positive or negative impact. When it comes to positive interaction, this happens through the application of practices focused on generating safe environments, where the caregiver provides comfort, through gentle handling and using tools that promote the development of functional capabilities minimizing the possibility of generating more physical and psychological effects in sick and healthy animals. In the same way, it is about promoting adequate and supervised treatment. On the contrary, a negative HAR develops in threatening, isolated, unsafe environments and with the application of painful practices by handlers, increasing the possibility of multiplying the effects on cattle (83, 131).

In contrast to dairy cows, beef cattle are commonly raised on rangelands where human interaction can be limited to preventive medicine, routine procedures, animal mobilization within the facility to the chute, corrals or barns, making beef indicus cattle more reactive than animals raised in dairy intensive systems (132, 133). Furthermore, research has shown that *Bos indicus* animals might have a more marked stress-related reaction –assessed through cortisol concentrations– to events such as restraint, weaning, or transport, than *Bos taurus* species (an average of 32.60 ± 0.66 ng/mL vs. 25.81 ± 0.76 ng/mL, respectively) (95).

The high reactivity of *Bos indicus* breeds and crossbred animals has also been related to a susceptibility to stress during handling. Marçal-Pedroza et al. (134) reported this in Holstein-Gyr cows in the milking parlor. An association was found between reactive cows with higher cortisol (between 7.23–12.15 ng/mL) and oxytocin levels (between 5.01–7.82 pg./mL) procedures that cattle might associate with fear and negative emotions, particularly because stockperson characteristics such as attitude and skill highly influences animal behavior (135).

For example, artificial insemination can be a stressful event for beef cattle. In Nellore heifers, Ceballos et al. (135) evaluated reactivity traits (e.g., frequency of kicking, jumping, mooing, lying, kneeling, balking, and attacking) and the stress response (cortisol) during insemination procedures to negative practices (hitting the gate against the animal’s body, hitting, or prodding the heifer with a wooden stick). High cortisol concentrations and undesirable behaviors were present in animals receiving aversive handling. Likewise, these animals showed a 25% increase in kicking, jumping, vocalizing, lying, and kneeling. Incidence of these events was also linked with a 10-fold decrease in the chance of becoming pregnant although the exact mechanisms behind this event is unclear (135). Behavioral responses such as escape attempts when using the squeeze chute are also common when cattle is handled by non-trained people (136). Moreover, Fordyce et al. (22) found that hornless Brahman cross cattle had higher temperament score than Shorthorn animals (5.54 vs. 4.44, respectively). This finding was similar to what was reported by Chase et al. (137) when comparing the temperament of weaned calves from three different breeds: Angus, Brahman, Romosinuano, and their crossbred combinations. The authors found that Brahman calves had excitable temperaments (assessed by exit velocity, chute and pen scores) than Angus and Romosinuano breeds (137), which might suggest that techniques currently applied to *Bos taurus* might not be as efficient in *Bos indicus* cattle.

When stockpeople are not trained to manage animals properly, they might resort to yelling and shouting, events that significantly affect livestock and activate stress-related axes (138). According to Pajor et al. (139) and Grandin (140), shouting and yelling at cows can be as harmful as the use of electric prods. In this sense, Grandin (2), has reported that loud sounds over 85 dB could elevate heart rate. This has been found in beef heifers who had higher heart rates when hearing human shouting (84.0–100.5 bpm) than the sound of metal clanging (74.4–86.7 bpm) (141). Wilson et al. (63) noticed that when a noisy truck pulls up in, it increases the number of cows that become reluctant to move. Pereira et al. (67) recorded the physiological response of crossbred dairy cows (Holstein-*Bos indicus*) after interactions with a milker in two different milking systems. Negative interactions such as hitting increased respiratory rate, surface temperature, and activity level. Vocalization and defecation were also increased.

Knowledge about the species and their adequate handling practices is essential to ensure a positive HAR. Therefore, training stockpeople is currently applied and promoted in livestock farming, with several studies addressing its benefits (101). Training stockpeople in good practices, even during simple procedures such as vaccination, has been proposed by Ceballos et al. (136) to improve cattle welfare. In their study, undesirable behaviors including jumping, balking or attacking were more frequent in animals handled by non-trained farmers. Likewise, the routine collection of data associated with the degree of animal welfare experienced in the herd (e.g., prevalence of skin lesions, mastitis or lameness) should be used to generate action plans in collaboration with farmers, regarding the critical points that can affect the comfort of dairy cows (142).

Programs have been generated that address aspects of attitude, norms, behavioral control and motivation in the implementation of controls with veterinary services and internal management, positively impacting HAR to minimize costs due to diseases and the prevalence of effects on udder health, including a higher SCC and increased prevalence of clinical and subclinical mastitis, and prevalence of injuries and bruises in dairy cattle (118, 119). Mastitis could be one of these injuries with estimated losses in various countries where the majority of *Bos indicus* cattle are developed, such as Ethiopia, with costs of up to 5.2% of the average milk income per cow (143). Training also influenced stockpeople attitude, showing higher percentages of positive attitudes from trained farmers than non-trained people (65.5% vs. 28.1%) (136). Similarly, the same authors reported in another study that frequent handling of beef Nellore and Nellore crossbreed cattle after 6 months reduced reactivity scores (from 4.20 ± 0.06 to 2.86 ± 0.05) and FS (from 1.76 ± 0.02 m/s to 1.14 ± 0.02 m/s) (133). This study showed that even in rotational grazing systems, constant interaction between humans and indicus cattle enhances positive HAR.

The negative behavioral responses, together with increases in cortisol (around 3.92 ± 1.20 ng/mL) when *Bos indicus* cattle is restrained in a squeeze chute, can decrease by about 1.67 ng/mL with habituation or repeated adequate handling of animals, as shown by Andrade et al. (144) in Brahman cattle. Moreover, Solano et al. (35) concluded that, when assessing the effect of HAR during husbandry procedures of *Bos indicus* cattle, it is essential to consider the social rank. In their study, cortisol concentrations of Brahman cows entering the handling chute were lower in low-ranking cows at the beginning of the experiment and at day 19, as a possible habituation to the procedure (6.3 ± 0.9 ng/mL and 0.5 ± 0.1 ng/mL). In contrast, high-ranking animals maintained higher cortisol levels (10.4 ± 2.8 ng/mL and 6.4 ± 1.2 ng/mL). Similarly, Macedo et al. (145) found higher cortisol concentrations (16.0 ± 2.1 ng/mL) in Nellore cattle when a negative HAR was present (e.g., rapid arm movements to frighten the animals, hitting or kicking them, twisting their tail). This result also affected the viability rate of embryos (19% lower than non-stressed cattle), which can affect the reproductive programs in the specie.

Habituation protocols of crossbred heifers (Holstein x Gyr) to reduce milking reactivity have shown to reduce flight distances (from 1.72 ± 1.22 m to 0.69 ± 0.52 m) and the number of steps (from 5.6 ± 7.73 to 3.37 ± 4.69) after a training protocol with human presence during 20 days (146). Similarly, Kamboj et al. (147) reported that pre-partum habituation of 15 days in primiparous Sahiwal cows reduced temperament scores (from 2.67 to 1.40), handling scores (from 2.40 to 1.13) and cortisol concentrations (16.11 to 5.61 ng/mL), showing that habituation to humans can reduce fear, stress, and reactivity of *Bos indicus* crossbred. This was also reported in Nellore cows after successive handling in the corral and containment chute (148). After 6 weeks of handling, reactivity score was lower than animals handled only twice (1.84 vs. 2.44), and it was found that the attitude of the handler influenced the behavioral response of animals.

In Brahman-crossbred heifers (Braford and Brahman x Angus) it was also reported that acclimation to handling resulted in a lower chute score (1.37 vs. 1.84) and cortisol concentrations (37.8 vs. 50.5 ng/mL) when compared to undisturbed heifers (149). According to Becker and Lobato (17), in their study carried out on *Bos indicus* crossed cattle in Brazil (forty Nelore × European male and female calves), to handle cattle in a gentle way, instead of the traditional aversive one procedures and habituation to management routines might reduce the occurrence of injuries to both livestock and handlers, reducing risks and losses for humans and improving the well-being of beef cattle.

**Figure 2:**
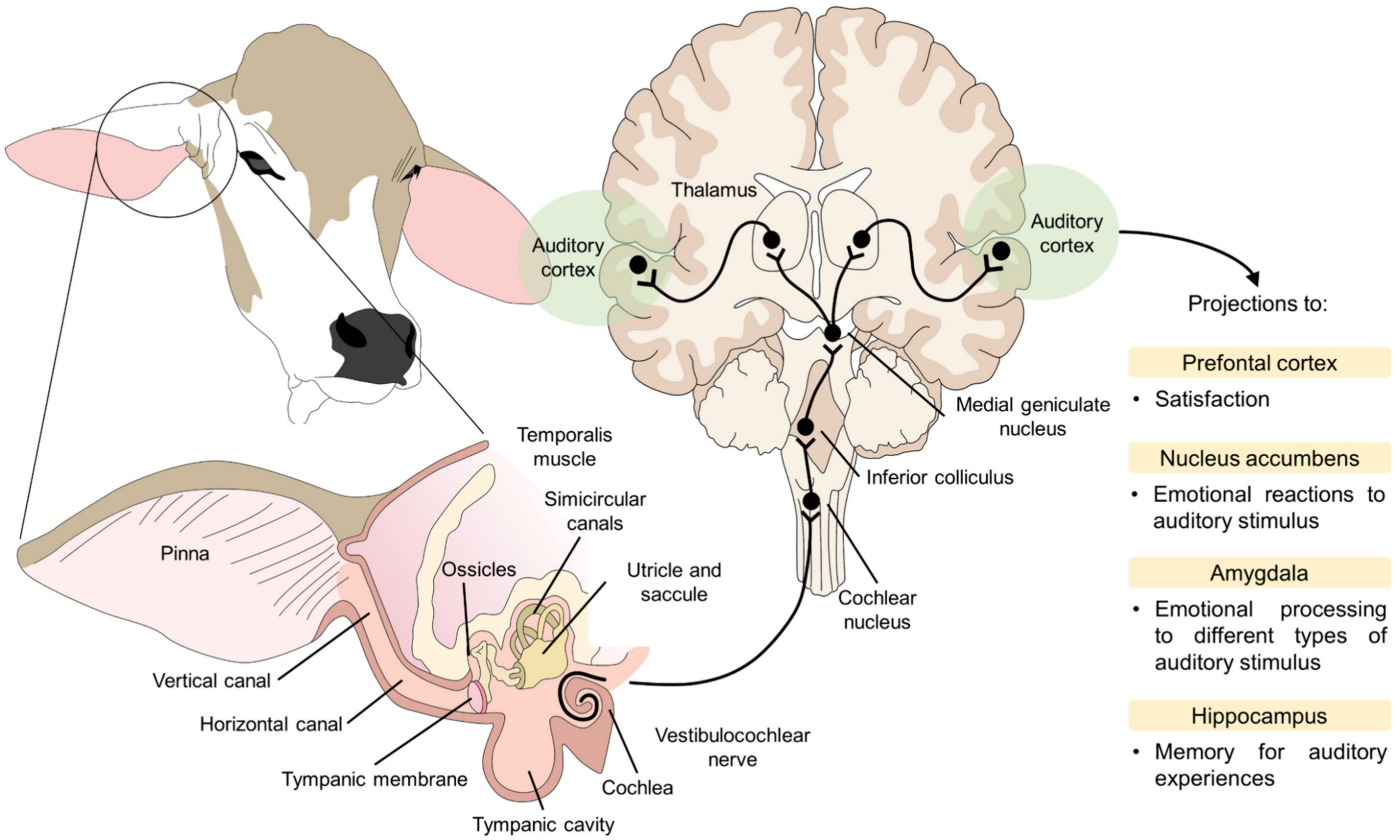
Auditory enrichment in dairy cattle. Auditory stimuli reach the auditory cortex through the vestibulocochlear nerve reaching the thalamus. Apart from recognizing the stimulus, the thalamus projects to other structures such as the amygdala, hippocampus, and prefrontal cortex to elicit the benefits that have been reported in dairy cattle, such as positive emotions when the stimulus reaches the amygdala.

Current management practices encourage the understanding of the natural behavioral repertoire of dairy cattle to improve their welfare. For example, long low-pitched sounds have been proposed to reduce noise disturbances in animals, including human voices, while Lange et al. (150) found that live soothing voices of humans and being stroked in the neck resulted in extended neck and ear postures reflecting a positive mental state. Using a normal tone promotes easy handling and prevents cortisol increases (76). Furthermore, when exposed to familiar sounds cattle can be stimulated to move to certain places throughout the facility (151). Figure 2 shows how the perception of positive auditory stimulation can cause positive emotional response in animals, particularly, in cattle, in whom sensory stimulation has been related to positive emotions and improved HAR (152–155).

In intensive dairy systems the interaction between animals and stockpeople is part of the daily routine of animals, the interaction with the milker and the type of milking system can influence the valence of the HAR. For example, Pereira et al. (67) determined in *Bos indicus* crossbred dairy cows that practices such as pushing and groping during milking is more frequent in the milking system “bucket at the bottom.” This can reflect a potentially negative HAR where animals defecate more frequently (38.40%) due to the aversive human handling (67). Likewise, steps and kicks are frequently observed in primiparous crossbreed cows (Holstein-Gyr) during milking and in the squeeze chute (156).

As previously mentioned, *Bos indicus* and *Bos indicus* crossbreed animals tend to be more reactive and temperamental than *Bos taurus* breeds (17). Nonetheless, this reaction can be reduced with constant handling and by providing a positive HAR (156). Moreover, the presence of the calf during milking and weaning could be considered beneficial when handling *Bos indicus* type cattle due to the highly maternal protective behavior of these breeds (157). In this sense, *Bos indicus* cattle tend to react when humans are less than 1 m from the calf, particularly within the first 30 days post calving (158).

Adopting natural behavior and reaction of *Bos indicus* breeds to current management practices could help to improve HAR. An example is grooming, a biological need of most farmed animals to clean themselves of mud, feces, insects, urine and ectoparasites by swatting the tail, licking, scratching with horns or hind feet, or even scratching on inanimate objects (159, 160). When this biological need is not covered, imbalances in animal welfare might be present and the lack of a positive stimulus to reinforce a positive mental state can cause detrimental effects on cattle (e.g., undesirable behaviors when not allowed to groom) (161). For this reason, enrichment for cattle may provide a way of encouraging grooming behavior (162–166).

**Figure 3:**
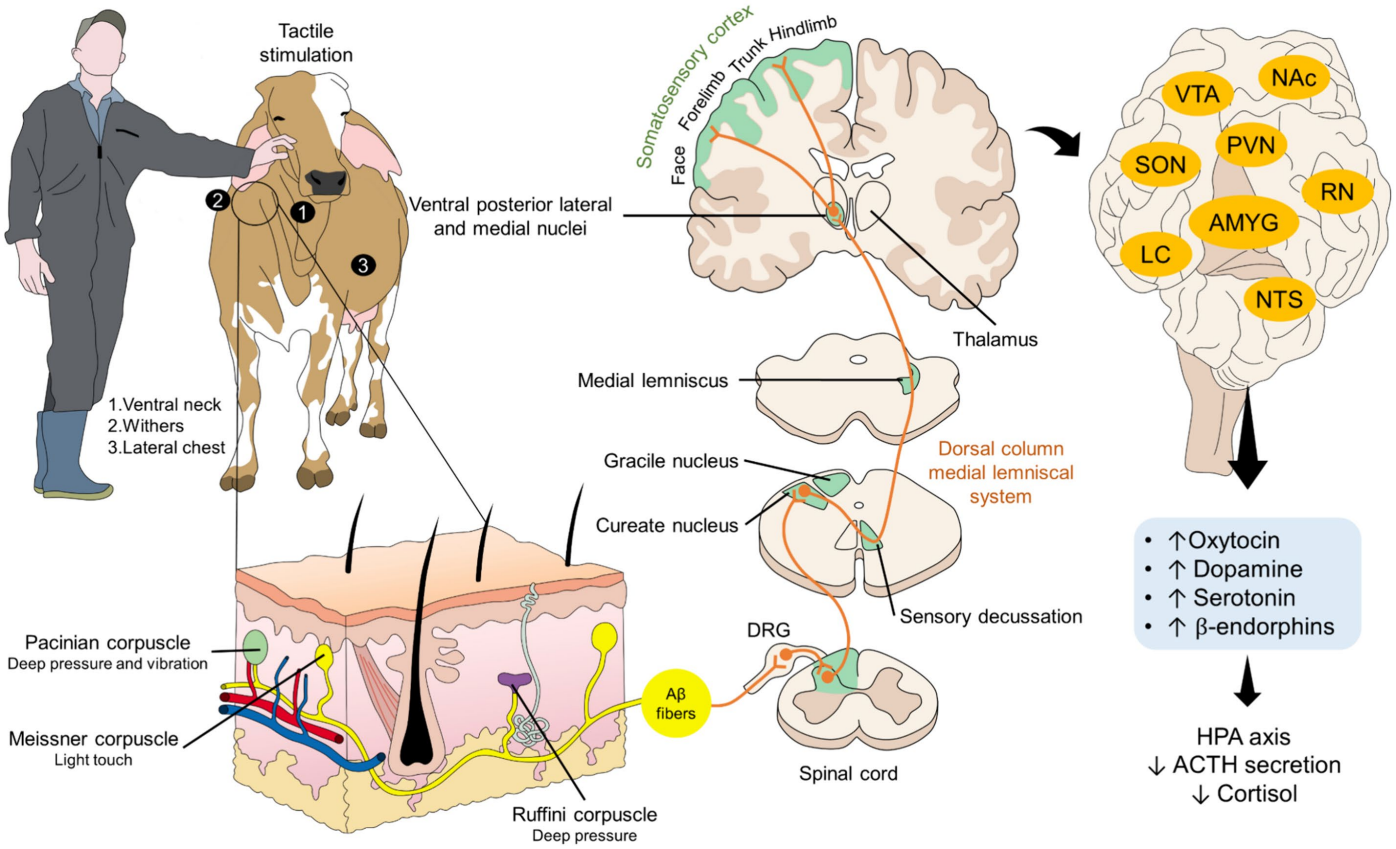
Cutaneous mechanoreceptors and their association with tactile stimulation in dairy cattle. Tactile stimulation in dairy cattle is often associated with positive outcomes such as the reduction of stress-related biomarkers (e.g., cortisol) and the increase in reward-or pleasure-related hormones such as oxytocin, dopamine, or serotonin. This is the result of the activation of low-threshold mechanoreceptors located in the skin, which respond to Aβ fibers. Through the dorsal column medial lemniscal system, tactile signaling reaches the somatosensory cortex and other cerebral structures such as the AMYG, NAc, VTA, SON, PVN, among others. AMYG, amygdala; ACTH, adrenocorticotropin; DRG, dorsal root ganglion; HPA, hypothalamic–pituitary–adrenal; LC, locus coeruleus; NAc, nucleus accumbens; NTS, nucleus of the solitary tract; PVN, paraventricular nucleus; RN, raphe nucleus; SON, supraoptic nucleus; VTA, ventral tegmental area.

Contact and tactile stimulation by stroking different parts of the cows’ bodies is regarded as a positive stimuli that is processed from the cutaneous mechanoreceptors activated with affective touch through brushing (Figure 3) (71, 155, 167, 168). Authors such as Schmied et al. (72), realized that the preferred areas for grooming by cows are the neck, dorsal and ventral parts. Although studies in *Bos taurus* animals are more prevalent, Ujita et al. (169) compared the effect of hand and brush tactile stimulation during the prepartum period of Gyr cows with non-stimulated animals. Using a score to assess the frequency of animal behavior such as stall, help, escape, tying strip, teat cups, and milking removal of teat cup in 40 cows, the authors found that positive tactile stimulation significantly decreased the score, meaning that fewer animals exhibited aversive behaviors. Furthermore, they also observed that cortisol decreased, and oxytocin increased in animals that received tactile stimulation. Similarly, Schmied et al. (72) reported that stroking the withers and the ventral part of the neck of 43 dairy cows for 3 weeks improved the reaction of the animals to rectal palpation. The authors observed that stroked animals had lower HR (decreases by 7.9 ± 4.1 bpm), in contrast to no-stroked subjects who recorded an increase in HR up to 10 ± 14.9 bpm.

These studies imply that gentle tactile stimulation decreases the level of fear or stress experienced by cattle during aversive events, particularly due to the increases of oxytocin, a hormone associated with positive emotions and mental state, impacting the fifth domain of the FDM. Similarly, brushing when animals are exposed to a common stressor such as the squeeze chute has showed benefits in Gyr dairy cows. In these animals, Ujita et al. (18) determined that pre-calving training animals to positive tactile stimulation for 14 days was effective by increasing the percentage of cows that calmly walked to exit the chute (56.6 to 96.0%). Moreover, the same animals had lower respiratory rate (35.22 ± 1.32 rpm vs. 31.52 ± 1.34 rpm) and rectal temperature (38.24 ± 0.10 rpm vs. 37.80 ± 0.11 rpm). A similar study made by the same authors found that pre-calving training of primiparous Gyr cows by gently brush them on the head, neck, trunk, udder, front legs and hind legs in the milking stalls reduced their protective behavior and moved less when humans approached the calf (170).

The implementation of training programs and continuous positive tactile stimulation that enhances a beneficial HAR during milking is important because *Bos indicus* breeds are known to have highly maternal instincts that can make them aggressive to handlers when not accustomed to the human-animal interaction (171). Adopting these types of strategies inside both beef and dairy farms could prevent affectations on the mental state of cattle.

## 5 Effects of HAR on the mental domain

The mental domain refers to the emotional state of the animals that is the results of both positive and negative experiences of *Bos indicus* cattle. The aforementioned Domains directly affect the mental state of animals, and this will trigger behavioral and emotional responses that are frequently seen when assessing HAR, such as fear (6).

Panksepp et al. (172) has extensively studied the effect that external factors such as a potentially negative HAR can be associated with a behavioral and adaptative response to an aversive stimulus. In Nellore cattle, even changes in the facial expression have been found in animals undergoing hot iron process (e.g., raised outer brow), changes that are associated to a painful stimulus (173). Likewise, in Nellore, Guzerat, and Gyr beef cattle, vigorous and abrupt head and tail movements were present in animals classified as highly temperamental (Guzerat had the highest average score 2.59 ± 1.33) (24). This is due to the interaction of brain structures such as the hippocampus, amygdala, and limbic system, triggering a freeze-and-flight response and also to the link between an aversive stimulus with an emotional response of severe fear (98).

### 5.1 Fear and avoidance

Dairy *Bos indicus* cattle are exposed to several situations during the milking process that might be stressful for the animals, such as the level of mechanization and even the milking system (67, 105). Some studies indicate a positive correlation between animals’ body condition and body composition (*r* = 0.8), an association that might help predict the disposition of adipose tissue and muscle tissue (174, 175). From a strictly physiological perspective, if we consider that the perception of acute fear can lead to the activation of the HPA axis, this event results in the secretion of glucocorticoids that facilitate the availability of energy resources (176). Moreover, it has been found that dairy cattle that experience fear increase the amount of residual milk due to the decrease in oxytocin secretion (177, 178). Thus, a negative HAR due to aversive handling during milking or herding using shouting or hitting could have a consequence on animal’s status.

Fear is a negative emotion that causes aversive physiological and behavioral changes, impairing the mental and physical health of cattle (179). It is behaviorally characterized by flight or freezing responses and can arise as a result of routine procedures such as preventive medicine, weighing, ear tagging, castration, dehorning, milking, and daily interaction with humans (98). Additionally, high intensity auditory, tactile, or visual stimuli are a common cause of fear (141).

Handler behavior such as shouting or an aggressive approaching to animals can be a major source of stress to cattle (70, 179). Human events such as jerky or abrupt movements, chasing animals, shouting, or prodding are all fear-inducing. These negative interactions simulate danger signals from a predator, establishing an association between humans, certain events, and fear (10).

**Figure 4:**
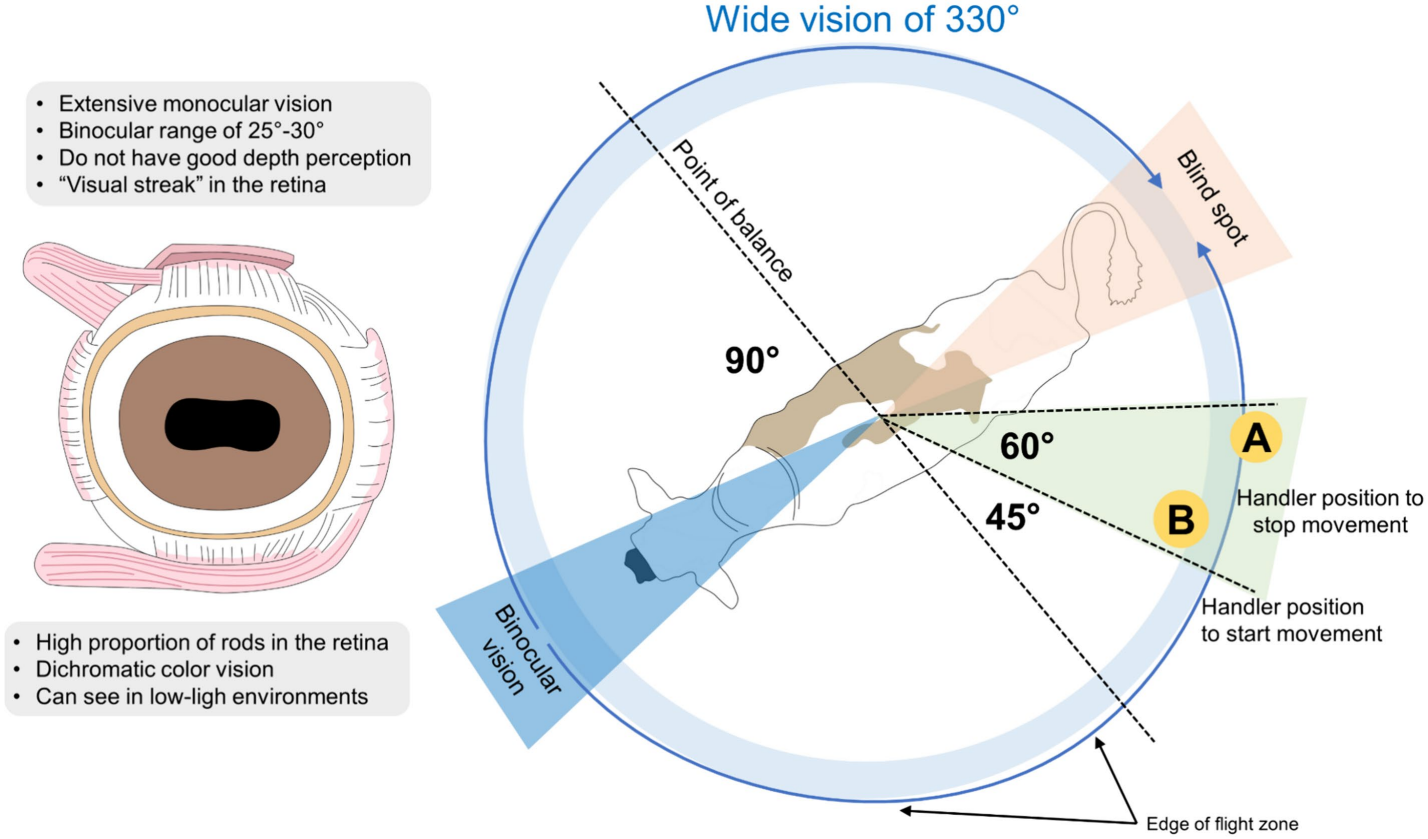
Visual acuity of dairy cattle and its effect on the flight zone.

Fear towards the stockperson or handler during a handling situation is usually assessed through escape-avoidance responses, or AD (180, 181). The AD is closely related to the flight zone, known as the distance, where a human can approach the animal before it moves away (182). When the handler or stockperson enters the flight zone, the animal will instinctively move away. In contrast, animals will stop moving when humans walk out from their flight zone (183). Working at the edge of the flight zone considers the 330° of vision of cattle and their region of best visual acuity at 130° to facilitate animal mobilization and handling. These characteristics are schematized in Figure 4, where considering the wide vision of cattle and the edge of the flight zone, handlers can take positions to facilitate the movement of the animals (at 45°) or to stop them (at 60°) (182, 184).

AD and flight zone depends on the species, breed, individual temperament, and previous experiences of animals. Hence, AD is a common method used to evaluate the quality and valence of HAR. Grandin (138) mentions that animals raised extensively can have a flight distance of up to 30 m, contrarily to cows reared in feedlot and dairy systems, where the animals tolerate human approach around 0 to 7.61 m. Nonetheless, training animals to become accustomed to human presence to reduce agitation, stress and fear (185). In particular, Murphey et al. (186) determined that *Bos indicus* cattle had higher flight distances (rank of up to 21), particularly Guzerat beef cattle, animals that were described as unapproachable by humans.

Gentle handling has been proposed as a strategy to improve HAR and reduce AD (17). In this sense, Probst et al. (187) evaluated 27 Limousin crossbred cattle exposed to gentle touching during 10 min for three non-consecutive days for 10 min. When considering voluntary approaches, the AV of enriched animals was significantly smaller compared to the control animals. Moreover, these animals reduced the frequency of avoidance behavior in the stunning box and their cortisol levels decreased, concluding that gentle and early interaction with humans reduces the level of fear. In other studies, gently handling completely prevented escape attempts in *Bos indicus* crossed beef calves, in contrast to non-handled animals, in whom 30% of attempts where observed (17). Likewise, aggressive behaviors were reduced to 5% and an interesting finding was that gently handled calves spent more time looking at the handler (33.63 ± 13.26 s), traits that suggest that habituation to handling even in *Bos indicus* animals can help to minimize the reactivity attributed to the species (17).

Stock people’s attitude is also important to reduce the level of fear that cattle might experience. In this way, positive attitudes aiming at gentle handling of the animals might help to establish an association between human presence and rewarding interactions (188, 189). Moreover, as shown in *Bos taurus* cattle, the implementation of tactile stimulation might improve management and reduce the fear associated with routine practices (4). On the other hand, an additional alternative is what was mentioned by Grignard et al. (190) that not only empathy and the type of management can exclusively influence the behavioral response, but the social environment can also affect the behavioral response during handling.

## 6 Future directions and strategies to promote positive human-animal interactions

Current farmers and animal handlers are aware of the importance of a positive HAR (191, 192). It has been reported that 21% of farmers believe that HAR generates fear in animals and this can affect productivity (61, 62). However, whilst there is some research, further research is needed to determine the impact of training and empathy level of the farmers on the level of fear that the animals show during handling. Additionally a factor that has been hypothesized but not fully explored is the relationship between the level of pain perceived by animals and negative interactions between stockpersons and animals (191–193), since fear can alter the pain threshold or might sensitize animals to pain, known as stress-induced hyperalgesia.

It is known that lateralization is widespread among livestock species, and can be defined as those behaviors including motor, sensory and cognition responses that are consistently biased to one side of the body (194). Future research should explore the methodological integration of laterality with ethological approaches for the evaluation of cow welfare during milking, allowing the generation of strategies to provide animals with choice around this, particularly in indicus breeds. This may give rise to technological interventions to ‘funnel’ animals to their preferred side through automation with consequent benefits for production, and animal welfare as well as human health and safety (195, 196). Provide housing and handling facilities where dairy cattle can be easily handled and moved can also prevent negative HAR and the use of aversive methods such as hitting them with sticks, prods, slaps, or shouting to move animals (109, 191, 192). When designing dairy cattle facilities, is essential to study the behavior of animals to provide adequate and safe environments for the animals that will also be reflected as positive HAR (110, 191, 192, 197).

Complementary techniques to evaluate fear-responses are part of the current precision livestock farming. For example, Stewart et al. (198) assessed exit speed and its effect of the ocular surface temperature in crossbred heifers subjected to two treatments: being hit with a plastic tube and being startled by a sudden movement of a plastic bag. The authors found that eye temperature decreased after both events at 20 and 40 s. This study did not only show that aversive practices elicit fear and can have physiological consequences for the animals, but also shows that infrared thermography could be applied to evaluate the welfare of dairy livestock.

Training in gentle handling has been shown to significantly decrease the incidence of handler injury (199). Positive reinforcement training also has benefits, leading to reduced avoidance behaviors when subjected to a subcutaneous injection, and consequently improving animal welfare and personnel safety (200). Finally, it is important to consider that high animal productivity is not always an indicator of welfare, and a multi-faceted assessment approach is required (191, 192, 197). Giving options and certain control of the situation to dairy cattle might be another way to improve the welfare of dairy animals. Dairy cattle generate preferences for entering the milking parlor and milking place, and when they are prevented from doing so as a result of excessive handling, inadequate milking design, or poor human-animal interaction, effects on production and physiological parameters such as total milk yield and heart rate may be recorded (117, 155, 191, 201, 202).

Some other lines of research could focus on new animal-based indicators, the non-invasive and practical analysis of the reactions to aversive or painful stimuli such as injuries or diseases in *Bos indicus* cattle with both dairy and meat objectives. The direct relationship with human-animal interaction considering that this type of livestock usually presents less interaction throughout its life with its handlers, for which monitoring of postures and facial expressions (203) during the post-castration or dehorning pain process, it would be very useful for identifying preventive and therapeutic measures to modify or implement (204). In addition to this, another line of research related to the physical health domain could be inclined towards the establishment of normal thermal parameters in various thermal windows (205) or anatomical regions (e.g., mammary gland) through the use of non-invasive tools such as infrared thermography (206) and its application during routine milking practices and activities where human-animal interaction is observed, analyzing, for example, the effect of the type of handler on the rates and prevalence of mastitis in *Bos indicus* cattle (207).

## 7 Conclusion

The literature shows that the temperament of *Bos indicus* cattle is determined not only by the genotype but also by interaction with the human environment. Therefore, it is essential to promote positive HAR when handling these breeds because *Bos indicus* animals are prone to be more reactive than *Bos taurus*, increasing the risk of eliciting negative physiological, endocrine, and behavioral responses (i.e., escaping attempts, fearfulness, or aggression), thus, increasing accident risk for both animals and handlers. The use of tactile stimulus or auditory stimulation are strategies that could help improve the HAR in both dairy and beef systems with *Bos indicus* animals.
